# Estimating prognosis in patients with acute myocardial infarction using personalized computational heart models

**DOI:** 10.1038/s41598-017-13635-2

**Published:** 2017-10-19

**Authors:** Hao Gao, Kenneth Mangion, David Carrick, Dirk Husmeier, Xiaoyu Luo, Colin Berry

**Affiliations:** 1 School of Mathematics and Statistics, University of Glasgow, Glasgow, UK; 2 British Heart Foundation, Glasgow Cardiovascular Research Centre, Institute of Cardiovascular and Medical Science, University of Glasgow, Glasgow, UK; 3 Golden Jubilee National Hospital, Clydebank, UK

## Abstract

Biomechanical computational models have potential prognostic utility in patients after an acute ST-segment–elevation myocardial infarction (STEMI). In a proof-of-concept study, we defined two groups (1) an acute STEMI group (n = 6, 83% male, age 54 ± 12 years) complicated by left ventricular (LV) systolic dysfunction; (2) an age- and sex- matched hyper-control group (n = 6, 83% male, age 46 ± 14 years), no prior history of cardiovascular disease and normal systolic blood pressure (SBP < 130 mmHg). Cardiac MRI was performed in the patients (2 days & 6 months post-STEMI) and the volunteers, and biomechanical heart models were synthesized for each subject. The candidate parameters included normalized active tension (*AT* ^norm^) and active tension at the resting sarcomere length (*T* ^req^, reflecting required contractility). Myocardial contractility was inversely determined from personalized heart models by matching CMR-imaged LV dynamics. Compared with controls, patients with recent STEMI exhibited increased LV wall active tension when normalized by SBP. We observed a linear relationship between *T* ^req^ 2 days post-MI and global longitudinal strain 6 months later (r = 0.86; p = 0.03). *T* ^req^ may be associated with changes in LV function in the longer term in STEMI patients complicated by LV dysfunction. Further studies seem warranted.

## Introduction

Death rates after acute ST-segment–elevation myocardial infarction (STEMI) have fallen markedly in recent years^1^, but the incidence of heart failure post-MI has remained persistently high^2^, and heart failure remains one of the most important causes of death post-MI^3^. Adverse left ventricular remodelling after acute MI portends an increased risk of heart failure^4,5^, based on the pathophysiology of impaired left ventricular systolic function^6,7^, infarct size^8^ and microvascular pathology^9,10^. Approximately one half of all patients who survive a STEMI have persistent, irreversible myocardial damage that is associated with a longer-term risk of heart failure^9,10^.

In clinical practice, therapeutic decisions are informed by an evidence base linked to surrogate biomarkers, notably left ventricular ejection fraction (LVEF)^1^. On the other hand, LVEF reflects changes in left ventricular dimensions during the cardiac cycle, rather than contractility (pump function), and the majority of patients who die prematurely have a normal or mildly reduced LVEF^11^. Therefore, on an individual patient basis, risk prediction using LVEF remains somewhat limited. Advances in myocardial strain imaging hold promise^12^. Clinicians usually lack information on left ventricular biomechanics in their patients after an acute STEMI, but even if biomechanics were described, their clinical significance in this setting is unknown. Personalized computational heart modelling have emerging potential to quantitatively assess biomechanical parameters which directly reflect left ventricular pump function^13,14^.

Biomechanical heart models have advanced from idealized theoretical concepts to subject-specific models with multi-scale i.e. intra-cellular, whole cell, inter-cellular tissue, organ, and multi-physics e.g. electrophysiology, myocardial biomechanics, blood flow, coupling under physiological and pathological conditions^14–16^, including post-MI^17,18^. Biomechanical models that are based on cardiac magnetic resonance (CMR) imaging, although complex and time-intensive, have potential to realise computational heart modelling for personalized risk prediction in the clinic^13^.

We designated candidate biomechanical parameters implicated in left ventricular pump function including $A{T}^{\text{nor}}$ which is the normalized active tension (*AT*) in the left ventricular wall, defined as $A{T}^{\text{nor}}=AT/(\text{Systolic}\,\text{Blood}\,\text{Pressure})$. $A{T}^{\text{nor}}$ reflects the active left ventricular tension associated with a 1 mmHg increase in systolic blood pressure (BP). We also focused on ${T}^{{\rm{req}}}$ which is the active myocyte tension at the resting sarcomere length when myocyte stretch is 1, which reflects the required contractility at that moment. ${T}^{{\rm{req}}}$ is therefore a parameter of global biomechanical function. We hypothesized that these novel biomechanical parameters would be (1) more discriminative for the assessment of individual patients with acute STEMI compared to healthy controls matched for age, and sex and, (2) potentially have greater prognostic significance for left ventricular function in the longer term. Our hypothesis was that these biomechanical parameters more closely reflect contractility and left ventricular pump performance than standard global measures, such as LVEF, and therefore would be discriminative.

There is a knowledge gap on the clinical significance of biomechanical parameters in patients following an acute STEMI. We aimed to investigate the discriminative value of candidate biomechanical indices estimated by computational heart modelling in patients with acute STEMI and healthy control subjects matched for age and sex. Left ventricular biomechanics *in vivo* were assessed using an immersed boundary-finite element (IB/FE) method incorporating data on myocardial function and infarct characteristics from the CMR scans obtained from patients with acute severe STEMI and healthy controls without any history of cardiovascular disease.

## Results

The characteristics of the study participants, including their CMR findings, are described in Table 1 and in the Supplement. The patients with acute STEMI had evidence of extensive infarction (40.5 ± 7.8% left ventricular mass) and microvascular obstruction (8.3 ± 4.5% left ventricular mass), as revealed by contrast-enhanced CMR. The mean LVEF was moderately reduced (41 ± 5%).

**Table 1:** Patient characteristics.

	Hyper-control	STEMI	p-value
*Characteristics*			
Age, years	46 ± 14	54 ± 12	0.3
Sex, male: female	5:1	5:1	1.0
Systolic blood pressure, mmHg	129 ± 13	114 ± 14	0.09
Diastolic blood pressure, mmHg	74 ± 14	74 ± 13	1.0
*Cardiac magnetic resonance*			
LV EDV, mL	121 ± 12	146.6 ± 27	0.06
LV ESV, mL	52 ± 10	96.1 ± 21.3	< 0.01
LVEF, %	57 ± 6	41 ± 5	< 0.01
GLS, %	−20.0 ± 5	−10.2 ± 3.2	< 0.01
Infarct size, % LV mass	~	40.5 ± 7.8	
Microvascular obstruction, n %		6 (100)	
Microvascular obstruction, % LV mass		8.3 ± 4.5	

EDV: end-diastolic volume (ml), ESV: end-systolic volume (ml), LV: left ventricular, LVEF: left ventricular ejection fraction (%), SBP: systolic blood pressure (cuff-measured) mmHg; GLS: global longitudinal strain; Infarct size is defined as a percentage of left ventricular mass. *p* values are reported using the student t-test by comparing the control groups to the STEMI group. Values are presented as mean ± standard deviation unless otherwise stated. *Detailed characteristics for the STEMI patients are provided in Supplementary Table S1.

**Figure 1:**
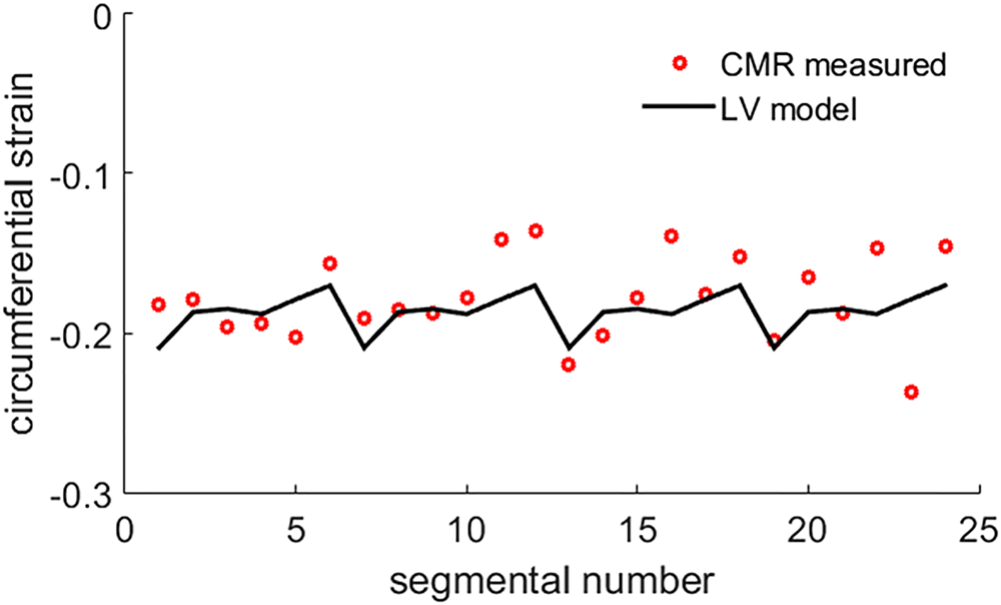
Predicted systolic strain from a personalized left ventricular model compared with *in vivo* measurements in one healthy subject.

**Figure 2:**
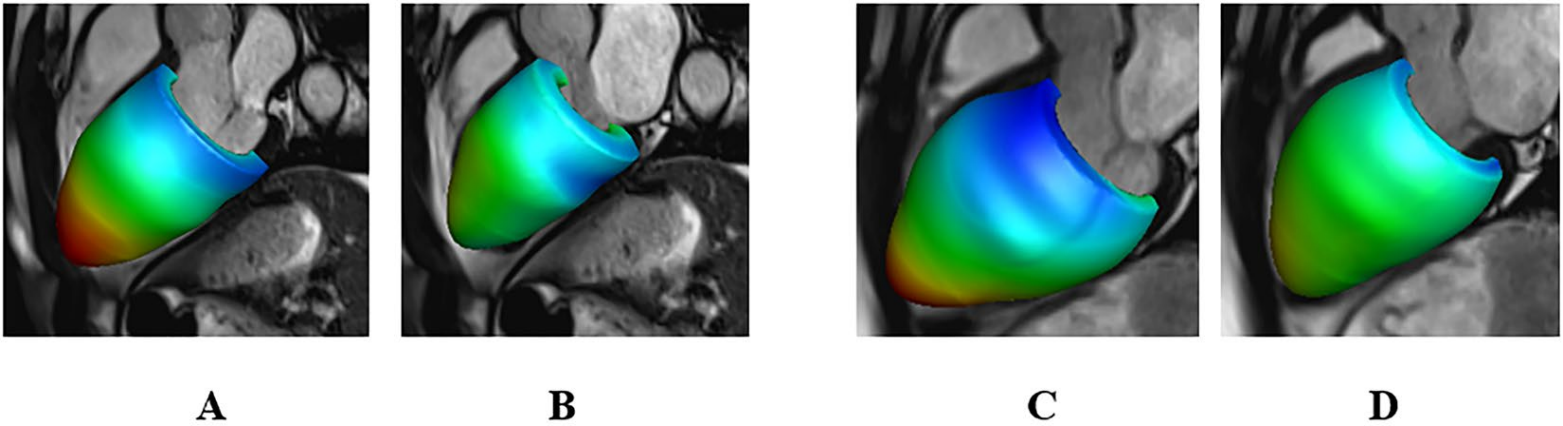
Simulated left ventricular dynamics in a healthy control subject and a patient after acute STEMI. (**A**,**B**) Deformed left ventricular geometries superimposed in CMR images at end-diastole and end-systole for the healthy control and for the MI patient (**C**,**D**). (**A**–**D**) are coloured by displacements related to the early-diastole geometry. Red: high, blue: low.

Table 2 summarizes left ventricular cavity volume and strain difference between the simulated values from personalized Left ventricular models and the *in vivo* CMR measurements. The relative difference of LV end-diastolic volume (LVEDV) in both the hyper-control group and the STEMI group were within in 2%. Systolic circumferential strain difference in the hyper-control group was slightly higher than the STEMI group, which was because the objective function in the hyper-control group incorporated left ventricular end-systolic volume (Eq. (8)). Figure 1 shows an example of systolic circumferential strains from a simulated left ventricular model after personalization, which matched well with the CMR measurements. In Fig. 2A and B, the simulated left ventricular geometries from one healthy control subject at end-diastole and end-systole are shown with superimposed CMR images. Similarly, the results from a patient are shown in Fig. 2C and D. The simulated left ventricular geometries agreed well with the CMR images.

**Table 2:** Optimization results in the hyper-control group and the STEMI group.

	ED volume difference (%)	ES volume difference (%)	Systolic strain difference (%)
Hyper-control	1.3 ± 1.3	2.2 ± 1.6	6.5 ± 5.1
STEMI	0.8 ± 0.7		1.1 ± 0.4

Volume difference was defined as $(\sum _{i=1}^{6}\frac{|{V}_{i}-{V}_{i}^{\text{measured}}|}{{V}_{i}^{\text{measured}}}\times 100{\rm{ \% }})/6$, and *V* is the simulated LV cavity volume, ${V}^{\text{measured}}$ is the measured value; strain difference was defined as $(\sum _{i=1}^{6}|\frac{{\bar{\varepsilon }}_{i}-{\bar{\varepsilon }}_{i}^{\text{measured}}}{{\bar{\varepsilon }}_{i}^{\text{measured}}}|\times 100{\rm{ \% }})/6$, and ${\bar{\varepsilon }}_{i}$ is the average systolic strain in one subject, which is ${\bar{\varepsilon }}_{i}=(\sum _{i}^{N}{\varepsilon }_{i})/N$, in which *N* is the total segmental number.

**Figure 3:**
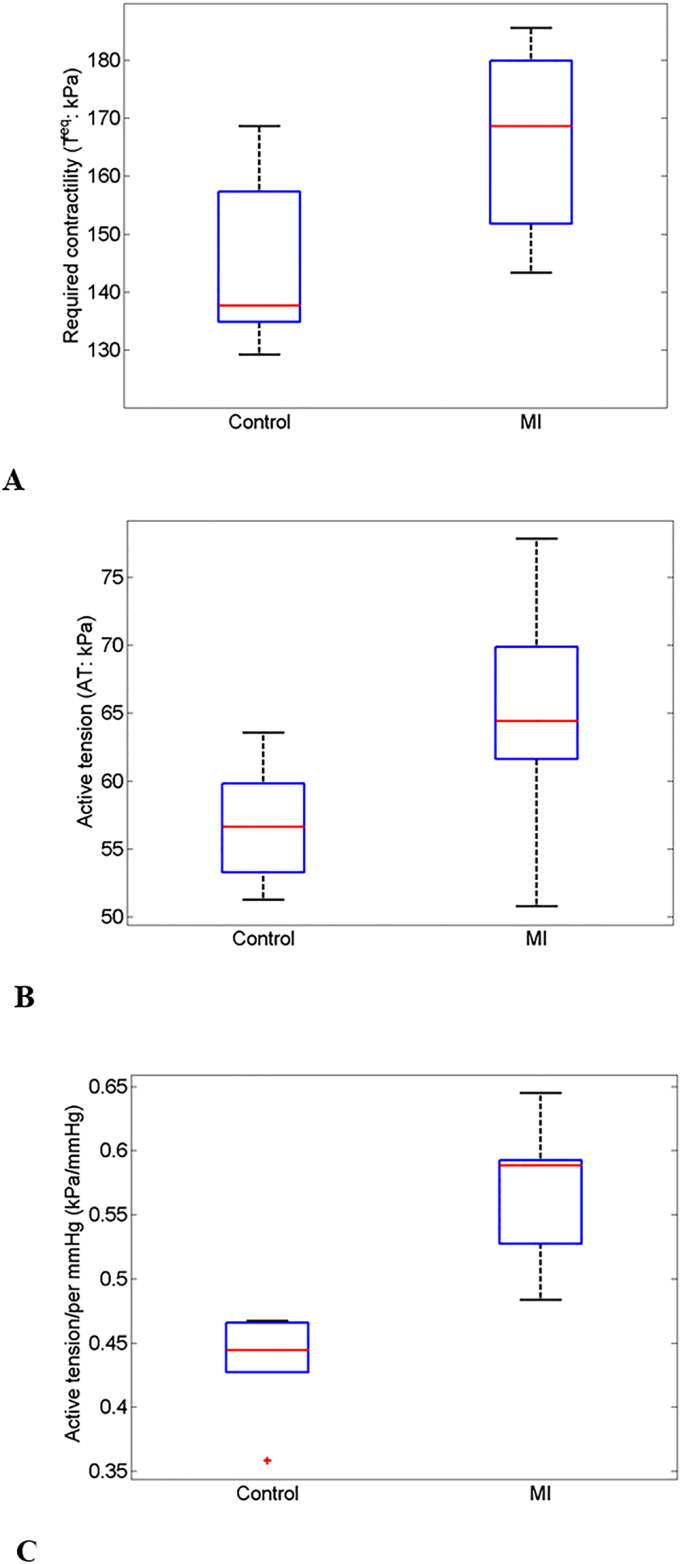
Comparisons between the control groups and the STEMI group in: (**A**), required contractility; (**B)** average systolic active tention; (**C**), systolic blood pressure; and (**D**), active tension generation per mmHg ventricular pressure increase.

The biomechanical parameters, myocardial active tension (${{\boldsymbol{\sigma }}}^{a}$) and required myocardial contractility (${T}^{{\rm{req}}}$), were normally distributed. The average ${T}^{{\rm{req}}}$ in the hyper-control group and the STEMI group is shown in Fig. 3. ${T}^{{\rm{req}}}$ in the hyper-control group was 144 ± 15 kPa, which was lower than the value of the STEMI group ((166 ± 18 kPa; *p* = 0.04), as shown in Fig. 3A. Compared with the hyper-control group, ${{\boldsymbol{\sigma }}}^{a}$ was slightly higher in the STEMI group (65 ± 9 kPa vs. 57 ± 4kPa; *p* = 0.08), as shown in Fig. 3B. Figure 3C compares the normalized active tension ($A{T}^{{\rm{nor}}}$), which was defined as ${{\boldsymbol{\sigma }}}^{a}$/systolic BP, were significantly lower in the control groups compared to the value in the STEMI group (0.43 ± 0.04 vs. 0.57 ± 0.06; *p* < 0.01). This difference may indicate that following an acute STEMI, higher active tension was necessary to maintain normal left ventricular pump function to compensate the loss of functional myocardium in MI region.

**Figure 4:**
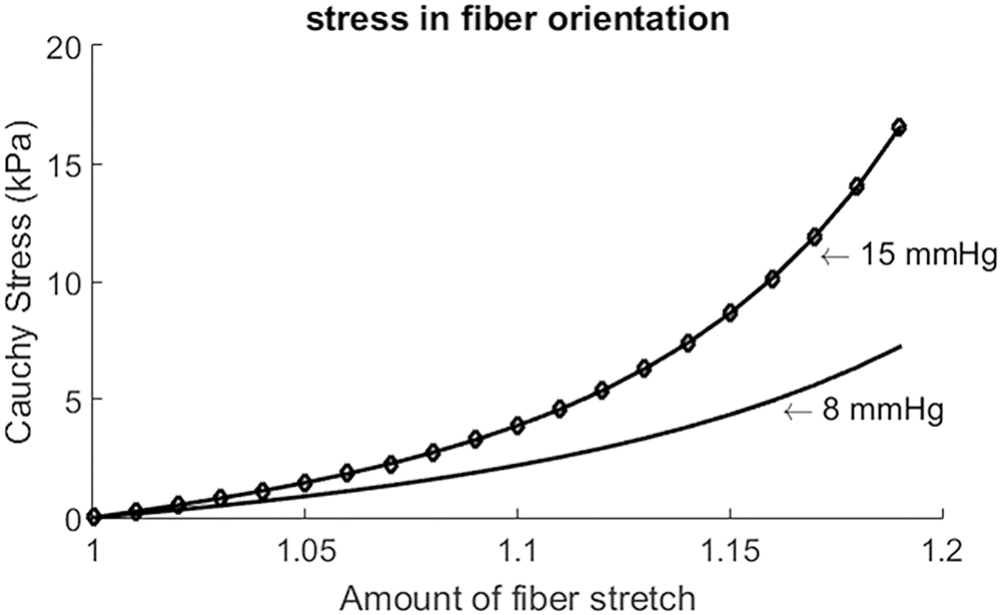
Estimated stiffness along myofibre direction under differnet end-diastolic pressure.

Since direct invasive measures of LVEDP were not available, these data were assumed based on typical reference values. We further increased LVEDP to be 15 mmHg in a healthy subject in order to estimate how different LVEDP values affect left ventricular systolic contraction. The estimated myofibre passive stiffness with 15 mmHg LVEDP was much higher compared to the estimated stiffness with 8 mmHg, as shown in Fig. 4. On the contrary, left ventricular systolic function was not affected much after rerunning the model following the personalization procedure. We found that ${T}^{{\rm{req}}}$ was decreased by 4%, while ${{\boldsymbol{\sigma }}}^{a}$ and $A{T}^{{\rm{nor}}}$ were nearly the same, with a mean difference of 0.5%. Therefore, ${T}^{{\rm{req}}}$, ${{\boldsymbol{\sigma }}}^{a}$ and $A{T}^{{\rm{nor}}}$ were overly not sensitive to changes in LVEDP if the left ventricular model matches volume and deformation at end-diastole. Indeed, similar findings were reported by Sun *et al*.^19^.

### Clinical correlates of the biomechanical parameters at baseline

In all healthy controls, only ${T}^{{\rm{req}}}$ was weakly related to weight and systolic BP (summarized in Supplement Table S2), but not related to age, height and sex. In the STEMI group, we observed associations between ${{\boldsymbol{\sigma }}}^{a}$ and systolic BP, height, between $A{T}^{{\rm{nor}}}$ and LVEF. We did not observe associations between ${T}^{{\rm{req}}}$, ${{\boldsymbol{\sigma }}}^{a}$, $A{T}^{{\rm{nor}}}$ and age, weight, sex, measured biochemical biomarkers including peak troponin I, C-reactive protein, and duration of symptoms (ischemic time) in the patient group at baseline.

### Associations between biomechanical parameters at baseline and left ventricular function at 6 months

**Figure 5:**
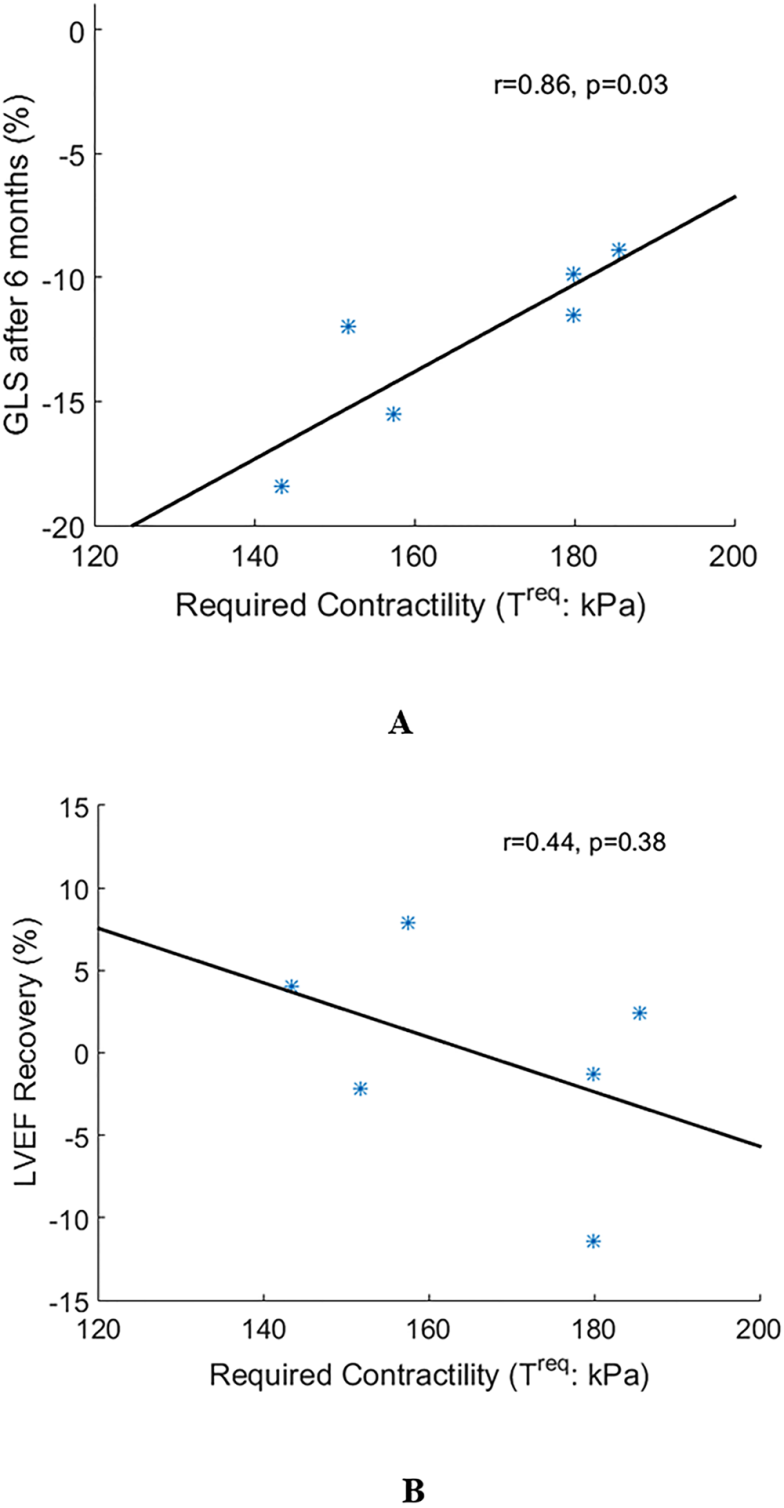
The scatter plots of (**A**): the global longitudinal strain (GLS) and (**B**): LVEF change after six months in the STEMI group in related to the required contractility.

Table 3 summarizes the correlations between the LVEF, global longitudinal strain (GLS), ${T}^{{\rm{req}}}$, ${{\boldsymbol{\sigma }}}^{a}$ and $A{T}^{{\rm{nor}}}$ at baseline and left ventricular function at 6 months in the STEMI group. We observed a strong linear relationship between ${T}^{{\rm{req}}}$ estimated 2 days post-STEMI and GLS at six months (r = 0.86; *p* = 0.03), as shown in Fig. 5A. This result indicates that a lower ${T}^{{\rm{req}}}$ after an acute STEMI complicated with no-reflow could have prognostic value for prediction of the recovery in left ventricular pump function (greater GLS) in the longer term. Figure 5B shows the relationship for change in LVEF (%) at six months from baseline after the acute STEMI and ${T}^{{\rm{req}}}$. We observed a weak negative correlation between ${T}^{{\rm{req}}}$ and changes in LVEF over time (r = −0.44, *p* = 0.38), however, the result was not statistically significant.

**Table 3:** Standard and novel biomechanical parameters early post-MI, and their relationships with LV systolic function at 6 months.

Baseline	Coefficient	Change in LVEF after six months		Global longitudinal strain after six months		
		95% confidence interval	p-value	Coefficient	95% confidence interval	p-value
LVEF, %	−0.65	[−0.96 0.33]	0.16	0.59	[−0.42 0.95]	0.22
GLS, %	−0.21	[−0.87 0.73]	0.70	0.42	[−0.59 0.92]	0.41
Required contractility (${T}^{\text{req}}$), kPa	−0.44	[−0.92 0.58]	0.38	0.86	[0.17 0.98]	0.03
Active tension (${{\boldsymbol{\sigma }}}^{a}$), kPa	0.76	[−0.14 0.97]	0.08	−0.40	[−0.91 0.61]	0.43
Normalized Active tension ($A{T}^{{\rm{nor}}}$), kPa/mmHg	0.79	[−0.06 0.98]	0.06	−0.69	[−0.96 0.28]	0.13

The normality test was performed by the One-Sample Kolmogorov-Smirnov Test, except for change in left ventricular ejection fraction (LVEF) at baseline, GLS, ${T}^{\text{req}}$, ${{\boldsymbol{\sigma }}}^{a}$, $A{T}^{{\rm{nor}}}$ at baseline and global longitudinal strain (GLS) at six months followed standard normal distributions, therefore, Pearson’s correlation analysis was adopted for the correlation analysis.

## Discussion

In this study, we modelled left ventricular dynamics at end-diastole and end-systole in patients following acute severe STEMI complicated by no-reflow and in healthy controls. The computational left ventricular models were exclusively based on *in vivo* CMR data along with detailed models of active myocardial tension generation. For the first time, we have shown that, compared with the hyper-control group, patients with recent STEMI complicated by left ventricular systolic dysfunction, exhibited increased active tension generation. The biomechanical parameters, required contractility (${T}^{{\rm{req}}}$) and normalized active tension ($A{T}^{{\rm{nor}}}$), were distinctly different between patients and controls. Furthermore, we have found preliminary evidence that these parameters may have prognostic value for left ventricular function at 6 months, whereas LVEF and global longitudinal strain did not (presumably reflecting the lower magnitude of correlation and the limited sample size of selected patients with relative large infarct size and left ventricular dysfunction). We have provided proof-of-concept insights into the potential clinical relevance of novel biomechanical parameters (${T}^{{\rm{req}}}$ and $A{T}^{{\rm{nor}}}$) of left ventricular pump function.

Most computational heart modelling nowadays is based on nonlinear soft tissue mechanics using finite element methods^15^. In this study, an immersed boundary approach was employed, which has been used extensively to model cardiac dynamics and valves accounting for blood-tissue interaction since its introduce in 1970s^20^. In recent years, the authors’ group has further developed a hybrid immersed boundary method to study left ventricular dynamics, which agreed well with *in vivo* CMR measurements^18^, commercial FEM packages^21^, and validated with other modelling approaches^22^.

To estimate passive material property, a population based LVEDP was assumed for the hyper-control group and the STEMI group based on the fact that LVEDP is generally higher in MI patients (10–25 mmHg) compared to healthy subjects (5–10 mmHg)^23^ as in our previous study^18^. In our model, myocardial systolic function ${T}^{{\rm{req}}}$ and $A{T}^{{\rm{nor}}}$ are not explicitly depended on passive parameters, but through ED volume, systolic circumferential strain and pressure. Therefore, the matched LVEDV and deformation at end-diastole helps to reduce the effects on systolic function caused by different ED pressures. Other studies^19^ also have shown that variations in passive parameters had little effects on estimated myocardial contractility. Therefore, the assumed LVEDP will not affect our conclusion on myocardial contractile function much in this study. However, myocardial passive stiffness is highly depended on the ED pressure, as shown in Fig. 4. Therefore, caution needs to be paid when comparing myocardial stiffness between the healthy controls and the MI patients because a higher LVEDP will indicate higher passive stiffness^24^. Future studies shall use measured LVEDP whenever possible so that diastolic left ventricular mechanics can be investigated more accurately and reliably.

Regional circumferential strains from cine images were estimated using in-house developed b-spline deformable registration method^25^ (Figure S1 in the supplementary material), in which segmentation of left ventricular wall boundaries and region definitions at end-diastole are the only user inputs. This approach was initially compared with Displacement Encoding with Stimulated Echoes (DENSE)^26^ in a small number of healthy volunteers and MI patients^25^, and validated within a large cohort of healthy volunteers^27^ recently. In this study, we further performed a reproducibility study (intra-/inter-observer variability) six months later. Details are provided in the supplement. In the healthy group, the relative mean bias was 0.4 ± 6.5% for intra-observer, and 0.3 ± 8.1% for inter-observer. Similar results were found in the MI group. The inter-correlation coefficient was > 0.93 for all analysis. This is consistent with findings from the study by Mangion *et al*.^27^, in which they demonstrated that cine-estimated circumferential strain using this b-spline deformable registration method has similar diagnostic performance as DENSE within healthy individuals, and more reproducible than feature tracking^27^. Similar as in other strain estimation techniques using MR imaging, the overall accuracy of our cine strain estimation approaches would be affected by imaging quality, out-of-plane motion, respiratory motion, etc.^28^.

In systolic phase, only ${T}^{{\rm{req}}}$ was inversely determined, while other parameters of the active contraction models were fixed. The same approach has been widely used in many other studies^16,17,19,29–31^ to model *in vivo* ventricular function, but avoid to determining many different parameters at cellular level, which is nearly impossible from *in vivo* CMR imaging. To evaluate how sensitive the modelled left ventricular contractile function was to uncertainties in strain measurements, simulations were rerun by varying measured strains with 10% change (subtracting 0.015 in magnitude). We found that a 10% decrease in average systolic circumferential strain would result in 20% decrease in optimized ${T}^{{\rm{req}}}$, however, the difference in $A{T}^{{\rm{nor}}}$ is less than 1%. This much higher sensitivity in ${T}^{{\rm{req}}}$ was not surprising because systolic circumferential strain is a key function reflecting myocardial contractility^32^, as reported by Sun *et al*.^19^. As discussed above, strain estimation from cine images has high reproducibility with nearly zero bias in intra-/inter observer variability in both the healthy and MI groups, and similar diagnostic performance as in DENSE^25,27^, and is well within the reported ranges from other studies^27,33–35^. Moreover, ${T}^{{\rm{req}}}$ is determined by multiple strain data (Eqs 8 and 9) and is further constrained by a matched end-systolic volume in healthy subjects (Eq. 8). Therefore, we believe in this ‘*extreme-case’*–‘*hyper-control’* study, uncertainties in circumferential strain estimation using cine images will not affect our findings on ${T}^{{\rm{req}}}$ and $A{T}^{{\rm{nor}}}$. However, further studies are required to investigate how ${T}^{{\rm{req}}}$ and $A{T}^{{\rm{nor}}}$ would be affected by various uncertainties within unselected patients, such as NSTEMI patients. Needless to say, accurate measurements (3D MR imaging) and access to a rich dataset (voxel-wise motion/strain data) are essential to further improve the credibility of cardiac models. Indeed, though not routinely available, 3D MR imaging are begun to be used in left ventricular modelling^16,36^.

Our results on myocardial contractile function after acute MI are consistent with prior mathematical modelling of animal and human hearts, in which myocardial contractility was shown to paradoxically increase in diseased hearts, potentially reflecting a compensatory response^18,36^. Chabiniok *et al*.^37^ found that in a pig model of reperfused MI, the estimated contractility in the remote zones increased progressively up to one-month later. We found that left ventricular contractility directly reflected by ${T}^{{\rm{req}}}$ in the group of STEMI patients was higher compared to the hyper-control group. Harding *et al*.^38^ found that the maximum myocardial contractility was not reduced in cardiomyocytes obtained from failing hearts compared to non-failing control hearts, rather reduced beta-adrenoceptor sensitivity was the distinguishing functional problem associated with clinical heart failure and increasing with age. Houser and Margulies^39^ further confirmed that the basal contractility was well preserved under resting conditions in dysfunctional hearts, but the ability to increase contractility (contractility reserve) in response to inotropic stimuli was severely depressed. The enhanced overall left ventricular contractility in the STEMI patients compared to controls in our study may be explained by adaptive mechanisms^6,40^, such as sympatho-adrenal and neurohumoral mechanisms, in order to preserve left ventricular pump function and cardiac output. Taken together, our results may provide insights into left ventricular biomechanics that complement the Frank-Starling law of the heart^7^. On the other hand, myocardial contractile reserve is finite and left ventricular remodelling in the longer term may have maladaptive consequences^7^. A cardiomyocyte with a lower ${T}^{{\rm{req}}}$ may have innate capacity to augment contractility, as shown in Fig. 5, supporting the possibility of recovery in left ventricular systolic function post-MI in patients with lower ${T}^{{\rm{req}}}$.

We have shown that biomechanical parameters of left ventricular function may present new insights into the pathophysiology of acute MI over and above standard measures of left ventricular pump function (Table 3). Our case-control study was designed by including groups of patients with left ventricular systolic dysfunction with similarly severe impairments of pump function, the insights gained in this study may not be generally applicable to all MI patients. Further research is warranted in order to assess the prognostic performance of ${T}^{{\rm{req}}}$ and $A{T}^{{\rm{nor}}}$ compared with other established measures of prognosis, such as LVEF and left ventricular end-systolic volume, in larger and less selected patient cohorts. Should this be the case then ${T}^{{\rm{req}}}$ and $A{T}^{{\rm{nor}}}$ may represent therapeutic targets in clinical research e.g. as biomarkers of treatment effect in experimental and clinical studies of novel therapies to prevent adverse remodelling.

The $A{T}^{{\rm{nor}}}$ was much lower in the hyper-control groups compared to the STEMI group. These results stimulate a new hypothesis based on biomechanics for the observed higher myocardial $A{T}^{{\rm{nor}}}$ in patients with recent STEMI. We hypothesize that contractility may be augmented in remote myocardium to generate sufficient active tension and preserve left ventricular pump function. This homeostatic adaptation would serve to compensate for the loss of functioning myocytes within the infarct zone. The requirement for enhanced and sustained active tension generation in the MI patients could lead to loss of contractile reserve that in turn could lead to impaired left ventricular systolic function and heart failure in the longer term. A diagram of the proposed hypothesis is illustrated in the Supplement (Figure S2).

Validation and verification of mathematical modelling remains a grand challenge in the whole biomechanics modelling communities. It is also an essential step for the success of the clinical translation. Growing efforts are being made through comparisons to experimental benchmark data^41^, to clinical images^19,42^ and to different computational models^21,22^. Directly measuring ${T}^{{\rm{req}}}$ and $A{T}^{{\rm{nor}}}$ can be extremely difficult *in vivo*, even *in vitro*. Moreover, *in vitro* measurements can differ significantly from *in vivo* assessments. For example, much higher Ca^2+^ sensitivity and isometric tension were reported in ‘intact’ myocytes than in ‘skinned’ myocytes^43^. Indirect validation can be achieved by comparing modelling results with other computational models. Table 4 summaries required myocardial contractility from recent computational ventricular models derived from *in vivo* clinical data. Although there are variations among different studies, required contractility in the healthy group and the STEMI group in our study are within the values reported by other studies. The variations might be due to inter-individual variations, sample size, different mathematical descriptions, etc. Furthermore, we also observed that estimated myocardial contractility is higher in diseased heart compared to healthy subjects (Table 4).

**Table 4:** Summary of estimated myocardial contractility from computational models of human hearts (Values for diseased hearts in bold fonts), HF: heart failure, MI: myocardial infarction, HV: healthy volunteer with normal cardiac function.

Studies	Imaging modality	Number of subjects	Myocardial contractility
Genet *et al*.^16^	Tagged MRI	5 HVs	143 kPa
Genet *et al*.^53^	3D cine, 3D tagged, 2D LGE MRI	1 MI patient	**146.9 kPa**
Wenk, *et al*.^17^	Tagged and LGE MRI	1 MI patient	**109.5 kPa**
Wang *et al*.^30^	Cine MRI	6 HVs	88 kPa (HV)
		5 hypertrophic HF	**160 kPa** (hypertrophic)
		9 non-ischemic HF	**124 kPa** (NI- HF)
Gao *et al*.^18^	Cine MRI	1 HV	168.6 kPa (HV)
		1 MI patient	**309.1 kPa** (MI)
Asner *et al*.^36^	Cine, 3D tagged, and 4D flow MRI	1 HV	139 kPa
Land *et al*.^43^	CT imaging	3 Patients with healthy heart function	120 kPa
Our study	Cine MRI	6 HVs	144 kPa
		6 STEMI patients	**166 kPa**

A comprehensive understanding of heart function includes physiology, pathology, biochemistry, biophysics, biomechanics, etc. One of the advantages of personalized mathematical models, such as the model that we have developed, has the potential to provide a unique way to understand quantitatively left ventricular pump function by integrating multiscale and multiphysics in one model^44^. LVEF and global longitudinal strain do not reflect contractile reserve (or effort) because these parameters are the final output of myocardial contraction, not the determinants of the contraction. However, the proposed biomechanical parameters (${T}^{{\rm{req}}}$ and $A{T}^{{\rm{nor}}}$) should not be considered as replacements of clinical indices, such as LVEF or global longitudinal strain. In this study, patients in the STEMI group had a relatively large size of infarction complicated by microvascular obstruction, thus, LVEF was enough to separate these individuals from healthy subjects, while biomechanical parameters, such as the ${T}^{{\rm{req}}}$, may further discriminated STEMI patients with similar LVEF as indicated in Fig. 5, a lower ${T}^{{\rm{req}}}$ may suggest that those patients could have better recovery in the longer term, different treatments might be possible. This observation suggests that ${T}^{{\rm{req}}}$ may provide additional incremental information above left ventricular pump function, and could potentially help the patient management. Well-designed experiments are needed to further explore the relationships between enhanced active tension and the loss of contractile reserve with measurements ranging from cellular contractility to tissue stress distribution, and their changes follow-up. Our proof-of-concept results are hypothesis generating and further studies of the associations between ${T}^{{\rm{req}}}$ and $A{T}^{{\rm{nor}}}$ with physiological and pathological biomarkers seem warranted.

### Other limitations

The required contractility parameter reflects the whole of the left ventricle, and regional values were not calculated. Although the sample size was limited with selected patients, it was similar to other studies involving computational heart modelling. As such, the results are hypothesis generating. The prognostic results provide preliminary validation. Other limitations include: (1) the “extreme case” design, which helped reduce the number for identifying the difference, however, it was still not clear to which extent the proposed biomechanical parameters can have prognostic values; (2) future work shall include different patient types, i.e. STEMI patients with preserved left ventricular function with/without ‘no-reflow’, and shall evaluate how biomechanical factors vary according to age and sex, and how they evolve after acute MI; (3) we only estimated the overall ${T}^{{\rm{req}}}$ in the functional myocardium, but not considering regional contractile function in the “border zone” and MI regions; (4) we have not explored other factors (i.e. different MI characteristics, such as intra myocardial hemorhage) that are known to affect left ventricular pump function and its recovery.

## Conclusion

In summary, we have simulated left ventricular dynamics using CMR imaging at end-diastole and end-systole for a group of patients with acute severe STEMI complicated by ‘no-reflow’ and an age-/sex-matched hyper-control group with normal blood pressure. Our results showed that the required contractility (${T}^{{\rm{req}}}$) in the STEMI group was higher than the hyper-control group, and that similar results were observed for the systolic active tension and normalized active tension ($A{T}^{{\rm{nor}}}$). ${T}^{{\rm{req}}}$ and $A{T}^{{\rm{nor}}}$ were not overly sensitive to end-diastolic pressure if the left ventricular model can match end-diastolic volume and deformation. Furthermore, on an individual patient basis, the enhanced contractility demand may have negative consequences for left ventricular function and remodelling post-MI within those patients, who had acute left ventricular systolic dysfunction due to large myocardial infarction and extensive microvascular obstruction. The differences in required contractility and normalized active tension between the healthy controls and the STEMI patients suggest that these novel biomechanical parameters may have clinical value for prognostication on an individual patient basis.

## Methods

### Study design

The UK Research Ethics Service (ethics committee references: 10/S0703/28 and 11/AL/0190) approved the study and all of the participants provided written informed consent. All methods, including MRI^45^, were performed in accordance with the relevant guidelines and regulations. Given the unknown validity of the parameters, and the potential to mistakenly reject (Type 1 error) or accept (Type 2 error) a true null hypothesis, we implemented an ‘*extreme-case’* – ‘*hyper-control’* design in a proof-of-concept study of the potential discriminative value of novel biomechanical parameters^46^.

### Study population: patients with acute STEMI

Patients were identified from the British Heart Foundation MR-MI study population (ClinicalTrials.gov identifier: NCT02072850). We identified six patients (*extreme case group*) with acute severe left ventricular dysfunction from a population of patients (n = 324) with acute STEMI who had been enrolled into a single centre prospective CMR cohort study between July 14, 2011 and November 22, 2012. Patients with acute STEMI were eligible for enrolment in this study if they had an indication for emergency PCI due to a history of symptoms consistent with acute myocardial ischemia and with supporting changes on the electrocardiogram (i.e. ST-segment elevation or new left bundle-branch block). Exclusion criteria represented standard contra-indications to contrast CMR, including a pacemaker and estimated glomerular filtration rate <30 ml/min/1.73 m^2^.

For extreme cases, we selected STEMI patients with acute severe left ventricular dysfunction due to “no-reflow”, which is a severe form of acute reperfusion injury^47^. “No-reflow”, which is defined as an acute reduction in myocardial blood flow despite a patent epicardial coronary artery, is independently associated with adverse remodelling and adverse outcome^48^.

### Control groups

We used community adverts and personal contacts to invite healthy volunteers to participate in an imaging study. We enrolled adults with no prior history of cardiovascular disease or treatment in whom cardiac disease was excluded based on a normal electrocardiogram (ECG) and a normal CMR scan. Six healthy volunteers were selected as the hyper-control group with normal office blood pressure (<130/80 mmHg) at the time of the scan per ESC Guidelines for the management of arterial hypertension^49^. The hyper-control group matched the acute STEMI group in age and sex.

### Measurement of blood pressure

Blood pressure (BP) was measured according to standard guidelines^50^. Subjects were invited to rest for ten or more minutes prior to the BP measurement. Two arm-cuff BP measurements were obtained in the sitting position at least 5 minutes apart. The standard size cuff was used and a larger or smaller bladder was used for large or thin arms, respectively. The cuff was placed at the level of the heart. We used auscultation of phase I and V (disappearance) Korotkoff sounds to identify systolic and diastolic BP, respectively.

### CMR imaging

CMR was performed at 1.5 Tesla (Siemens Avanto, Siemens Healthcare, Erlangen, Germany) in the six patients with acute STEMI 2 days after hospital admission and six months later. CMR was also performed in the healthy volunteers. Patients and healthy volunteers underwent the same imaging protocol except that healthy volunteers <45 years did not receive intravenous gadolinium contrast.

The CMR protocol for imaging left ventricular structure and functional assessment involved steady-state free precession cine scans with a short-axis left ventricular stack from the base to the apex. The slice thickness was 7 mm with 3mm gap, typical cine imaging parameters were matrix = 180 × 256, flip angle = 80°, TR: 3.3 ms, TE: 1.2ms, bandwidth: 930 Hz/pixel. The voxel size was 1.32 × 1.32 × 7 mm^3^. Cine images were acquired in the three-chamber, horizontal long-axis, and vertical long-axis planes.

In the STEMI group, late gadolinium enhancement (LGE) images covering the entire left ventricle were acquired 10–15 minutes after an intravenous injection of 0.15 mmol/kg of gadoterate meglumine (Gd2 + -DOTA, Dotarem, Guebert S.A.) using segmented phase-sensitive inversion recovery (PSIR) turbo fast low-angle shot sequence. Typical LGE imaging parameters were matrix = 192 × 256, flip angle = 25°, TE = 3.36 ms, bandwidth = 130 Hz/pixel, echo spacing = 8.7 ms and trigger pulse = 2. The voxel size was 1.32 × 1.32 × 8 mm^3^. Inversion times were individually adjusted to optimize nulling of apparently normal myocardium (typical values, 200 to 300 ms).

Left ventricular outcomes were assessed at 6 months using the same CMR protocol. We assessed change in LVEF (%) at six months from baseline after the acute STEMI and global longitudiual systolic strain at six months. Diogenes feature-tracking software (TomTec Imaging Systems, Germany) was used to quantify systolic strain from long axis cine images (horizontal, vertical long axis and left ventricular outflow tract cine images) in related to end-diastole, then these three longitudinal systolic strains were averaged to derive global longitudinal strain (GLS).

### Mathematical modelling of the left ventricle

**Figure 6:**
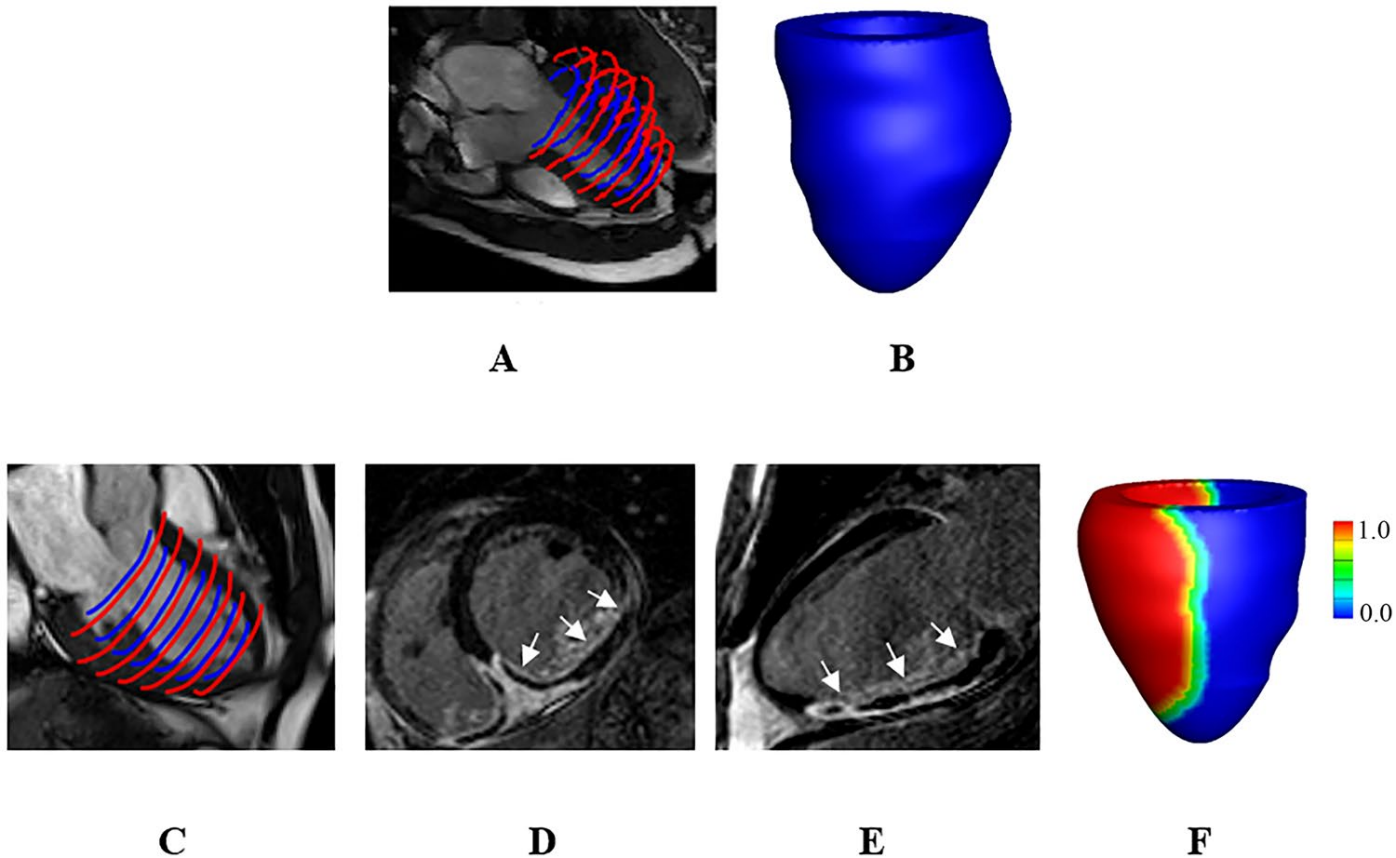
An example of the left ventricular model construction for a healthy control: (**A**), Left ventricular boundary segmentation; (**B**), reconstructed left ventricular geometry. An example of a diseased left ventricular model construction: (**C**), left ventricular boundary segmentation; (**D**), a LGE image in a middle short-axial position, MI region is indicated by the arrows, the black region inside the infarct region represents the microvascular obstruction. (**E**), a LGE image in a long-axial view; (**F**), reconstructed left ventricular model, the infart region is represented by the red colour, and the remote viable myocardium is represented by the blue color. A linear transition region is defined from the infart region towards the remote viable myocardium within 10 mm (1: 100% infarction, 0: viable myocardium).

Left ventricular geometries were reconstructed from *in vivo* cine CMR images in early diastole when left ventricular pressure is lowest, as shown in Fig. 6. In the STEMI group, short-/long- axial views of LGE images (Fig. 6D and E) were combined with cine images to define MI regions in the three-dimensional left ventricular models (Fig. 6F). Details of the computational heart model construction have been previously described^18^.

Left ventricular dynamics at end-diastole and end-systole were solved using an in-house developed IB/FE method^51^, which employs a finite element description of the myocardial mechanics that enables the use of realistic constitutive models. The formulation of the IB/FE approach and application in left ventricular dynamics can be found in refs^18,21^.

The total myocardial stress was defined as

1 $${\boldsymbol{\sigma }}=-p{\mathbb{I}}+\mu [\nabla {\bf{u}}+{(\nabla {\bf{u}})}^{T}]+\{\begin{array}{cc}{{\boldsymbol{\sigma }}}^{s} & {\rm{myocardium}}\\ 0 & {\rm{blood}}\,{\rm{region}}\end{array},$$

where $p$ is the blood pressure, $\mu$ is the blood viscosity, **u** is the fluid velocity, I is the identity matrix, and ${{\boldsymbol{\sigma }}}^{s}$ is the elastic myocardial stress, which was described as the sum of the passive stress (${{\boldsymbol{\sigma }}}^{p}$) and the active stress (${{\boldsymbol{\sigma }}}^{a}$). ${{\boldsymbol{\sigma }}}^{p}$ was determined using a strain energy function *W* ^18^,

2 $$W=\frac{a}{2b}\exp [b({I}_{1}-3)]+\sum _{i=f,s}\frac{{a}_{i}}{2{b}_{i}}(\exp [{b}_{i}{({I}_{4i}-1)}^{2}]-1)+\frac{{a}_{{\rm{f}}{\rm{s}}}}{2{b}_{{\rm{f}}{\rm{s}}}}(\exp [{b}_{{\rm{f}}{\rm{s}}}{({I}_{8{\rm{f}}{\rm{s}}})}^{2}]-1),$$

in which $a,b,{a}_{\text{f}},{b}_{\text{f}},{a}_{\text{s}},{b}_{\text{s}},{a}_{\text{fs}},{b}_{\text{fs}}$ are 8 unknown constitutive parameters, ${I}_{1}={\rm{t}}{\rm{r}}{\rm{a}}{\rm{c}}{\rm{e}}\,({\rm{C}})={\rm{t}}{\rm{r}}{\rm{a}}{\rm{c}}{\rm{e}}\,({{\mathbb{F}}}^{T}{\mathbb{F}})$ is the first strain invariant, ${\mathbb{F}}$ is the structural deformation gradient. The left ventricular myofibre architecture was described in terms of the myofiber direction ${{\bf{f}}}_{0}$ and the sheet axis ${{\bf{s}}}_{0}$, defined in the reference configuration. Based on these material axes, the following strain invariants were introduced as

3 $${I}_{{\rm{4f}}}={{\bf{f}}}_{0}\,\cdot \,({\rm{C}}{{\bf{f}}}_{0}),{I}_{{\rm{4s}}}={{\bf{s}}}_{0}\,\cdot \,({\rm{C}}{{\bf{s}}}_{0}),{I}_{{\rm{8fs}}}={{\bf{f}}}_{0}\,\cdot \,({\rm{C}}{{\bf{s}}}_{0}),$$

Assuming the fibre can only bear the loading when it is stretched, the terms involving ${I}_{{\rm{4f}}}$ and ${I}_{{\rm{4s}}}$ are non-zero only if ${I}_{4i} > 1$, then in the adopted IB/FE approach, the passive structural stress tensor (${{\boldsymbol{\sigma }}}^{p}$) was defined as

4 $${{\boldsymbol{\sigma }}}^{p}={\rm{\det }}{({\mathbb{F}})}^{-1}\frac{\partial W}{\partial {\mathbb{F}}}{{\mathbb{F}}}^{T}-\frac{{\beta }_{s}}{J}\,\mathrm{log}({J}^{2}),$$

where ${\rm{\det }}()$ is the determination of the deformation gradient, ${\beta }_{s}$ is a constant to further enforce the incompressibility of the, and $J={\rm{\det }}({\mathbb{F}})$.

The active stress ${{\boldsymbol{\sigma }}}^{a}$ was defined as

5 $${{\boldsymbol{\sigma }}}^{a}=T{\bf{f}}\otimes {\bf{f}}=T({\rm{time}},{{\rm{Ca}}}^{2+},{\rm{SL}},{T}^{{\rm{req}}}){\bf{f}}\otimes {\bf{f}}$$

where $T$ is the myocardial active tension, modelled by a group of ordinary differential equations of time, intracellular calcium concentration (${{\rm{Ca}}}^{2+}$), sarcomere length (${\rm{SL}}$), and the active tension ${T}^{{\rm{req}}}$ when the stretch of sarcomere is 1 (the so-called required contractility), **f** is the myofibre orientation at current configuration. ${T}^{{\rm{req}}}$ reflects the overall left ventricular contractility associated with the measured left ventricular pump function at the time of the CMR scan, but does not represent the maximum myocardial contractility which should be the contractility when myocardium is under maximum activation, for example under stressed CMR imaging. The difference between ${T}^{{\rm{req}}}$ and the maximum capacity of myocardial contractility would indicate the contractile reserve, which reflects how much myocardium can further increases its contractile function when needed.

A detailed description of the active tension model has been previously described in a prior study^18^. In brief, the contractile force *T* in Eq.(5) was modelled by cross-bridge cycling between the thin filaments and thick filaments, and calculated from a fading memory model^52^

6 $$T={T}^{{\rm{req}}}(1+{\beta }_{0}({\lambda }_{f}-1))\frac{z}{{z}_{{\rm{\max }}}}\times \{\begin{array}{ll}\frac{1+\alpha {\sum }_{i=1}^{3}{Q}_{i}}{1-{\sum }_{i=1}^{3}{Q}_{i}} & {\rm{if}}\,{\sum }_{i=1}^{3}{Q}_{i} < 0,\\ \frac{1+(2+\alpha ){\sum }_{i=1}^{3}{Q}_{i}}{1+{\sum }_{i=1}^{3}{Q}_{i}} & \text{otherwise},\end{array}$$

where *z* is the fraction of actin binding sites, ${z}_{{\rm{\max }}}$ is the maximum fraction of actin binding sites available at a given stretch ${\lambda }_{f}$, ${\beta }_{0}$ is a constant, *α* is a measure of the curvature of the force-velocity relation, ${Q}_{i}(i=1,2,3)$ were determined by

7 $$\frac{d{Q}_{i}}{dt}={A}_{i}\frac{d{\lambda }_{f}}{dt}-{\alpha }_{i}{Q}_{i},$$

where *A*_*i*_ and *α*_*i*_ are constant parameters.

### Boundary Conditions

The longitudinal displacement of the left ventricular base and the circumferential displacement of the basal plane were set to zero values. A pressure loading condition consistent with end-diastole was applied to the endocardial wall and the pressure consistent with systole was then rapidly ramped up. At the same time, a prescribed intracellular calcium transient was applied to the myocardium to induce active tension generation, which remained constant when the maximum value was attained. Because invasive left ventricular pressure measurements were not technically feasible at the time of the CMR scan in our hospital, a typical end-diastolic pressure of 8 mmHg was assigned to the hyper-control group and an elevated value of 16 mmHg was adopted for the STEMI group as in our prior study^18^. The peak systolic blood pressure was approximated from the brachial arm-cuff BP measurements. A detailed description of the IB/FE left ventricular model implementation can be found in ref.^18^.

### Model personalization

To personalize the left ventricular models, material parameters were determined by minimizing the difference between the simulated left ventricular dynamics and *in vivo* CMR measurements, including $a,b,{a}_{\text{f}},{b}_{\text{f}},{a}_{\text{s}},{b}_{\text{s}},{a}_{\text{fs}},{b}_{\text{fs}}$ in Eq. (2) for myocardial passive property and ${T}^{{\rm{req}}}$ in active tension generation (Eq. (5)), other active tension parameters were kept the same as in ref.^18^ Specifically, passive myocardial parameters ($a,b,{a}_{\text{f}},{b}_{\text{f}},{a}_{\text{s}},{b}_{\text{s}},{a}_{\text{fs}},{b}_{\text{fs}}$) in healthy hearts were inversely determined by matching the simulated left ventricular dynamics in diastole to the measured *in vivo* data (left ventricular cavity volume and regional circumferential strain estimated from cine images in related to end-diastole), the same approach as in our prior study^24^. To estimate ${T}^{{\rm{req}}}$ in healthy hearts, the left ventricular model was firstly inflated to the end-diastolic pressure with optimized passive parameters, then followed by the systolic contraction. ${T}^{{\rm{req}}}$ was determined by minimizing an objective function defined asin which ${{\varepsilon }_{i}}^{\text{'}}$ is the segmental systolic circumferential strain estimated from cine images using an in-house developed b-spline methods^25,27^, ${\varepsilon }_{i}$ is the corresponding strain from the simulated left ventricular model, *N* is the total number of the segments from short-axis images, $V^{\prime}$ is the measured left ventricular cavity volume from CMR images, $V$ is the simulated value.

8 $${{\rm{Obj}}}^{{\rm{HV}}}=\frac{\sum _{i=1}^{N}{({\varepsilon }_{i}-{\varepsilon }_{i}^{^{\prime} })}^{2}}{N}+{(\frac{V-V^{\prime} }{V^{\prime} })}^{2},$$

In the MI models, we assumed that the passive response of the infarct zone was 50 times stiffer than hypo- or akinetic myocardium which did not exhibit late gadolinium enhancement (i.e. potentially viable myocardium), as previously described^17,31^. The passive response for myocardium remote from the infarct zone, i.e. remote zone myocardium, was determined using a similar approach as used in the healthy left ventricular models. In systole, the infarct zone was modelled as a purely passive material, and ${T}^{{\rm{req}}}$ in remote zone was determined by matching the measured end-systolic circumferential strain data

9 $${{\rm{Obj}}}^{{\rm{MI}}}=\frac{\sum _{i=1}^{{N}_{{\rm{un}}}}{({\varepsilon }_{i}-{\varepsilon }_{i}^{^{\prime} })}^{2}}{{N}_{{\rm{un}}}},$$

where ${N}_{{\rm{un}}}$ is the number of segmental remote regions. The average value of ${N}_{{\rm{un}}}$ is $13\pm 3$. Note that the end-systolic volume and the strain (contractility) in the infarct zone were not used because of the purely passive response assumption of the MI region. Previous studies have shown that the uncertainty from estimates of passive stiffness arising from the population-based end-diastolic pressure has less effect on the contractility (${T}^{\text{req}}$) estimation^19^. Therefore, our estimates of left ventricular contractility may be more robust than the estimates of passive myocardial stiffness.

### Statistical Analysis

Given the unknown validity of the biomechanical parameters, we adopted an extreme case-control design^46^, and because of the computational complexity of modelling left ventricular biomechanics, the sample size was necessarily limited in order to be computationally efficient. Based on the study from Wang *et al*.^30^, the mean difference in the average required myocardial contractility (${T}^{{\rm{req}}}$) between the normal group and the left ventricular remodelled group was 72 kPa, and the square root of the average of the two standard deviations was 36 kPa. On the assumption of independent additive normal noise, a sample size calculation for a paired comparison of ${T}^{{\rm{req}}}$ using a two-sided t-test indicated that 6 subjects with complete data would confer 88% power at a significance level 0.05. The sample size calculation was performed with R statistical software. This sample size is similar to one other study using computational left ventricular modelling^29^.

Data distribution and normality was assessed using the Kolmogorov-Smirnov test, and data with a normal distribution were expressed as means together with standard deviation. Differences between the groups were assessed using two-sample t-test. Linear correlations were assessed using Person’s correlation. The statistical analyses were performed using MATLAB (Mathworks, MA, US) and a p-value ≤ 0.05 was taken as statistically significant.

## Electronic supplementary material

Supplemental Material
