# Supplementary material for: Estimating prognosis in patients with acute myocardial infarction using personalized computational heart models
Source: Sci Rep. 2017 Oct 19;7:13527. doi: 10.1038/s41598-017-13635-2 (PMC5648923; doi:10.1038/s41598-017-13635-2)
Supplement: Supplementary file 1 — Supplemental Material [file 41598_2017_13635_MOESM1_ESM.pdf]

Title: Estimating prognosis in patients with acute myocardial infarction using personalized computational heart models.

Authors: Hao Gao[1] PhD, Kenneth Mangion[2,3] MD, David Carrick[2, 3] MB ChB PhD, Dirk Husmeier[1] PhD, Xiaoyu Luo[1] PhD, Colin Berry[2,3] MB ChB PhD

Supplement Material

1. Supplement Table

Table S1. Clinical and cardiac magnetic resonance findings in the patients with acute STEMI.

Characteristics	
Height, cm	170 ± 4
Weight, kg	69.6 ± 12.5
Heart rate, bpm	73 ± 19
Medical history, n	
Previous angina	none
Previous MI	1
Previous PCI	1
Hypertension	3
Hypercholesterolemia	3
Current smoking	5
Body mass index, kgm^{-2}	24 ± 5
Body surface area, m^2	1.80 ± 0.13
CMR findings at baseline	
LV ejection fraction, %	41±5

LVEDV index, mL/m^2	91.7 ± 20.7
LVESV index, mL/m^2	54.3 ± 13.3
LV mass index, g/m^2	80.9 ± 22.7
Area at risk, % LV mass	48.6 ± 7.1
Infarct size, % LV mass	40.5 ± 7.8
Microvascular obstruction, % LV mass	8.3 ± 4.5
Follow-up CMR at 6 months	
Myocardial salvage, % LV mass	18.9 ± 6.7
Myocardial salvage index , % LV mass	39.1 ± 14.5
Change in LV ejection fraction at 6 months from baseline, %	-0.1 ± 6.6

Abbreviations: LV – left ventricular; LVEDV – left ventricular end-diastolic volume; LVESV – left ventricular end-systolic volume; MI – myocardial infarction; PCI – percutaneous coronary intervention. Body surface area is calculated using the Du Bois formula.

Table S2. Associations between mechanical factors and age, weight, height, sex, systolic blood pressure (SBP), left ventricular ejection fraction (LVEF), and global longitudinal strain (GLS) at baseline.

	Age	Weight	Height	Sex	SBP	LVEF	GLS
Hyper-control							
T^{req}	0.94(0.06)	0.39(0.45)	0.007(0.99)	-0.42(0.4)	0.79(0.06)	-0.62(0.17)	0.20(0.69)
$\boldsymbol{\sigma}^{a}$	0.44(0.39)	-0.14(0.80)	-0.37(0.47)	-0.63(0.18)	0.53(0.28)	-0.24(0.64)	-0.08(0.88)
AT^{nor}	-0.37(0.47)	-0.78(0.07)	-0.66(0.15)	-0.40(0.44)	-0.41(0.42)	-0.37(0.45)	0.33(0.52)
STEMI							
T^{req}	-0.56(0.25)	-0.26(0.61)	-0.24(0.65)	-0.38(0.46)	0.06(0.91)	0.47(0.35)	0.66(0.16)
$\boldsymbol{\sigma}^{a}$	0.16(0.77)	-0.35(0.49)	0.92(0.01)	-0.11(0.83)	0.76(0.08)	-0.34(0.51)	0.08(0.88)
AT^{nor}	0.34(0.51)	-0.32(0.54)	0.17(0.75)	0.38(0.46)	-0.21(0.69)	-0.77(0.07)	-0.71(0.12)

2. Supplement Figure

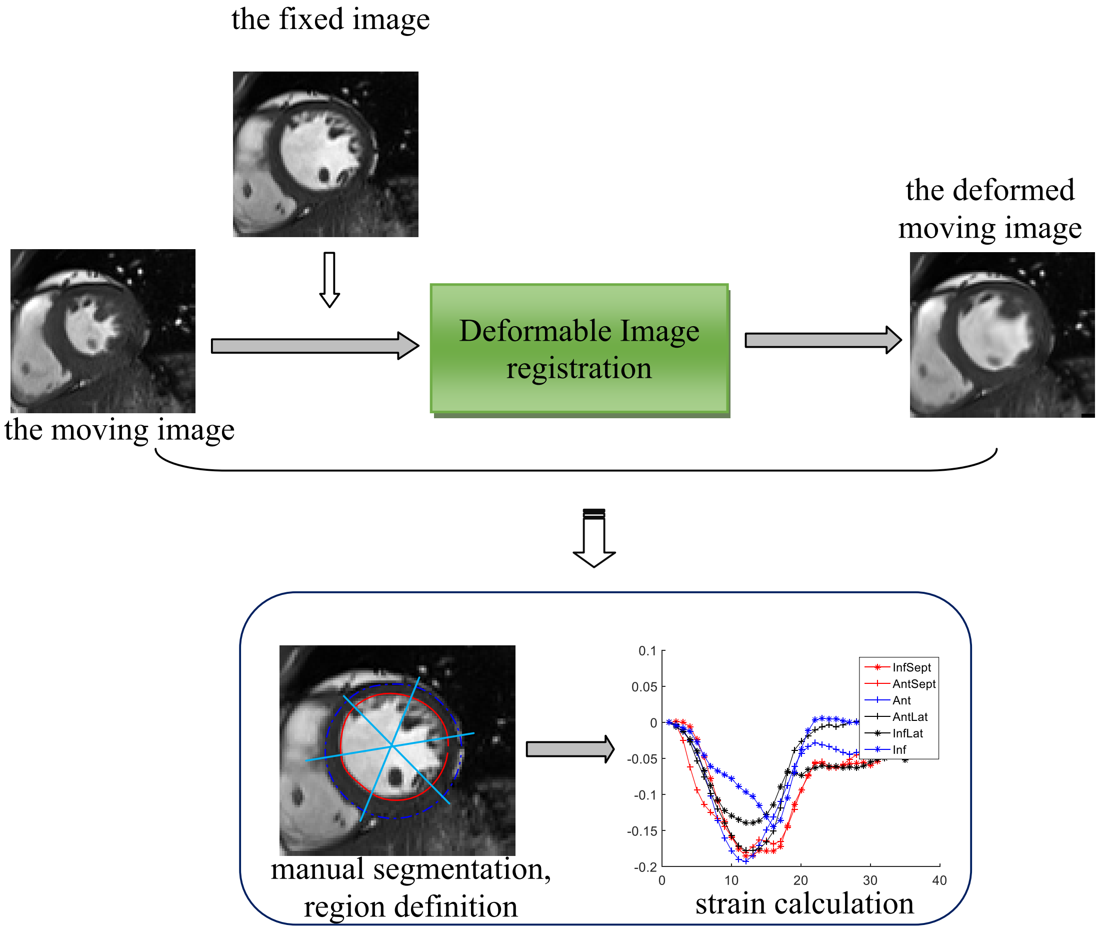

Figure S1. Schematic illustration of circumferential strain estimation from cine images using b-spline deformable image registration approach. The moving image is deformed according to the fixed image by minimizing the difference (i.e. pixel intensity) between the fixed image and the deformed moving image. In the next step, the endocardial and epicardial boundaries are first manually segmented, then segmental regions are defined based on the left ventricular (LV) and right ventricular (RV) insertions according to American Heart Association 17-segment definition. Finally the segmental strains are calculated based on the deformation fields from the deformation registration procedure. LV wall boundary segmentation and region definition are the only user inputs.

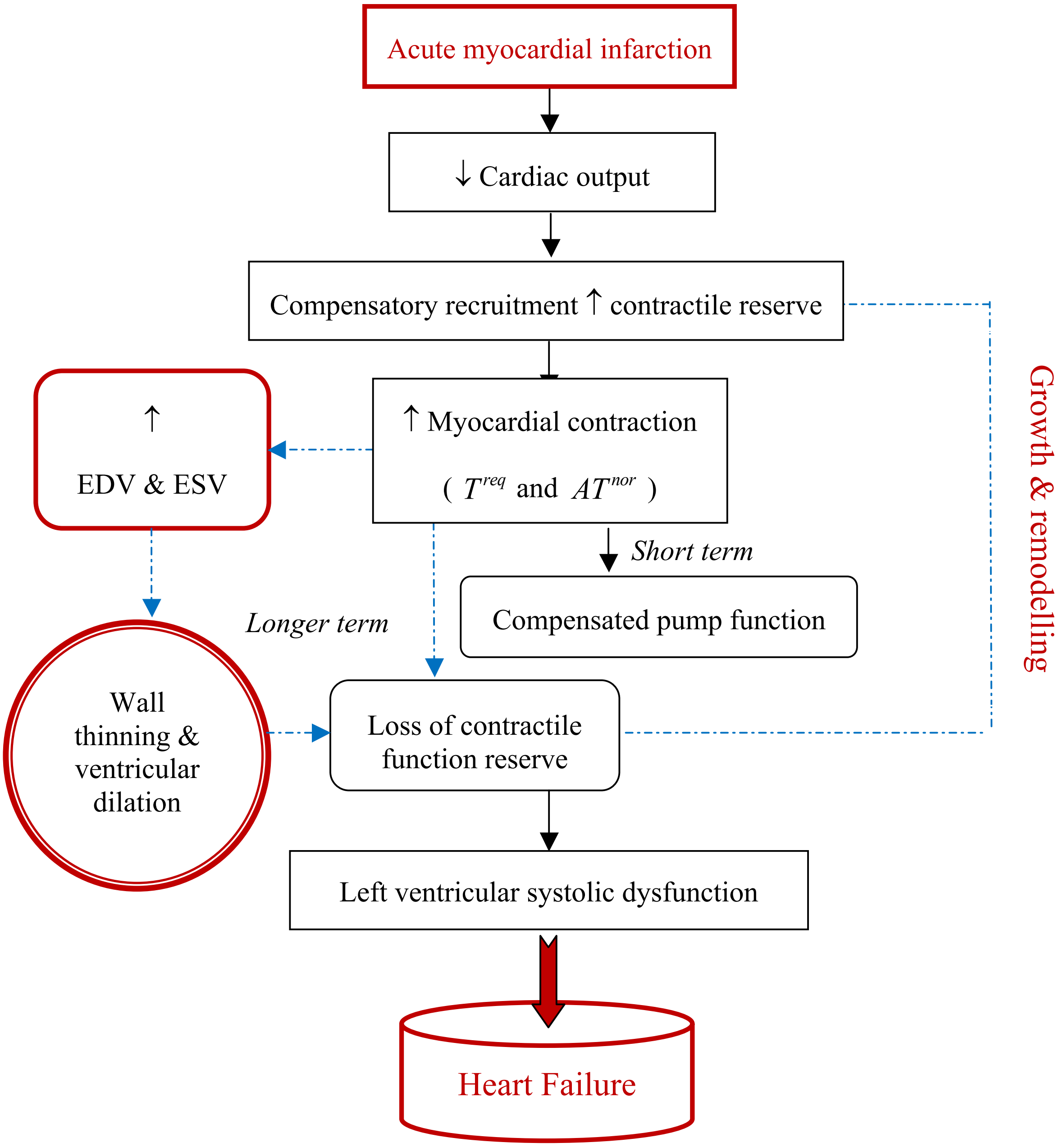

Figure S2. Schematic representation of the pathway of the proposed biomechanical factors in relation to adaptive changes in left ventricular pump function and remodelling after acute myocardial infarction. The schematic helps to expand on the role of biomechanical factors (inotropy) within the Frank-Starling law of the heart.

3. Supplement: Reproducibility Study on Cine Strain Estimation

To assess the reproducibility of cine strain estimation from the in-house developed b-spline approach, we re-estimated circumferential strains from cine images for both the healthy and MI groups. Cine images were reanalysed 6 months later, by the same operator (H.G.) for intra-observer variability, and by L.Y.F (from the authors' group) for inter-observer variability, who was trained before reanalysing. The results are summarized in the table below.

	Mean bias ±SD		ICC	
	HV	MI	HV	MI
Intra-observer	0.4±6.5%	0.4±6.8%	0.95	0.97
Inter-observer	0.3±8.1%	0.9±7.4%	0.93	0.96

HV: healthy volunteer, MI: myocardial infarction, ICC: inter-correlation coefficient, SD: standard deviation. Mean bias±SD is calculated as $\frac{\text{mean bias}}{\text{average strain}} \times 100\% \pm \frac{\text{SD}}{\text{average strain}} \times 100\%$.
